# Beliefs, Barriers, and Preferences of European Overweight Women to Adopt a Healthier Lifestyle in Pregnancy to Minimize Risk of Developing Gestational Diabetes Mellitus: An Explorative Study

**DOI:** 10.1155/2016/3435791

**Published:** 2016-01-14

**Authors:** Judith G. M. Jelsma, Karen M. van Leeuwen, Nicolette Oostdam, Christopher Bunn, David Simmons, Gernot Desoye, Rosa Corcoy, Juan M. Adelantado, Alexandra Kautzky-Willer, Jürgen Harreiter, Frans Andre van Assche, Roland Devlieger, Dirk Timmerman, David Hill, Peter Damm, Elisabeth R. Mathiesen, Ewa Wender-Ozegowska, Agnieszka Zawiejska, Pablo Rebollo, Annunziata Lapolla, Maria G. Dalfrà, Stefano del Prato, Alessandra Bertolotto, Fidelma Dunne, Dorte M. Jensen, Lise Lotte T. Andersen, Frank J. Snoek, Mireille N. M. van Poppel

**Affiliations:** ^1^Department of Public and Occupational Health, EMGO^+^-Institute for Health and Care Research, VU University Medical Centre, 1081 BT Amsterdam, Netherlands; ^2^Department of Health Sciences, Faculty of Earth & Life Sciences, EMGO^+^-Institute for Health and Care Research, VU University, 1081 BT Amsterdam, Netherlands; ^3^Institute of Health and Wellbeing, University of Glasgow, Glasgow G12 8RZ, UK; ^4^Institute of Metabolic Science, Addenbrooke's Hospital, Cambridge CB2 0QQ, UK; ^5^Macarthur Clinical School, Western Sydney University, Campbelltown, NSW 2751, Australia; ^6^Department of Obstetrics and Gynecology, Medical University of Graz, 8036 Graz, Austria; ^7^Institut de Recerca de l'Hospital de la Santa Creu i Sant Pau, 08026 Barcelona, Spain; ^8^CIBER Bioengineering, Biomaterials and Nanotechnology, Institute of Health Carlos III, 28029 Madrid, Spain; ^9^Divison of Endocrinology and Metabolism, Department of Medicine III, Medical University of Vienna, 1010 Vienna, Austria; ^10^KU Leuven Department of Development and Regeneration: Pregnancy, Fetus and Neonate, Gynaecology and Obstetrics, University Hospitals Leuven, 3000 Leuven, Belgium; ^11^Recherche en Santé Lawson SA, 9552 St. Gallen, Switzerland; ^12^Center for Pregnant Women with Diabetes, Departments of Endocrinology and Obstetrics, Rigshospitalet, Faculty of Health Sciences, University of Copenhagen, 1165 Copenhagen, Denmark; ^13^Medical Faculty I, Poznan University of Medical Sciences, 61-701 Poznan, Poland; ^14^BAP Health Outcomes Research SL, 33010 Oviedo, Spain; ^15^Universita degli Studi di Padova, 35122 Padova, Italy; ^16^Università di Pisa, 56126 Pisa, Italy; ^17^National University of Ireland, Galway, Ireland; ^18^Departments of Endocrinology and Gynecology & Obstetrics, Odense University Hospital, 5000 Odense, Denmark; ^19^Faculty of Health Science, University of Southern Denmark, 5230 Odense, Denmark; ^20^Department of Medical Psychology, EMGO^+^-Institute for Health and Care Research, VU University Medical Centre, 1081 BT Amsterdam, Netherlands; ^21^Department of Medical Psychology, Academic Medical Centre, 1105 AZ Amsterdam, Netherlands; ^22^Institute of Sport Science, University of Graz, 8010 Graz, Austria

## Abstract

*Introduction*. We explored beliefs, perceived barriers, and preferences regarding lifestyle changes among overweight European pregnant women to help inform the development of future lifestyle interventions in the prevention of gestational diabetes mellitus.* Methods*. An explorative mixed methods, two-staged study was conducted to gather information from pregnant European women (BMI ≥ 25 kg/m^2^). In three European countries 21 interviews were conducted, followed by 71 questionnaires in six other European countries. Content analysis and descriptive and chi-square statistics were applied (*p* < 0.05).* Results*. Women preferred to obtain detailed information about their personal risk. The health of their baby was a major motivating factor. Perceived barriers for physical activity included pregnancy-specific issues such as tiredness and experiencing physical complaints. Insufficient time was a barrier more frequently reported by women with children. Abstaining from snacking was identified as a challenge for the majority of women, especially for those without children. Women preferred to obtain support from their partner, as well as health professionals and valued flexible lifestyle programs.* Conclusions*. Healthcare professionals need to inform overweight pregnant women about their personal risk, discuss lifestyle modification, and assist in weight management. Lifestyle programs should be tailored to the individual, taking into account barriers experienced by overweight first-time mothers and multipara women.

## 1. Introduction

Gestational diabetes mellitus (GDM), which is defined as “carbohydrate intolerance resulting in hyperglycaemia of variable severity with onset or first recognition during pregnancy” [1], is a serious condition affecting 2–6% of pregnancies in Europe [2]. It adversely affects health outcomes for both mother and child in pregnancy and in their future health [3–6], with a sevenfold increased risk for the mother [7, 8] and eightfold increased risk for the offspring [9] of developing type 2 diabetes mellitus.

High maternal weight is associated with a substantially higher risk of GDM [10] and the prevalence for GDM continues to increase with the worldwide rise of obesity [11]. This suggests that prevention of GDM especially in the obese population is extremely important for both mother and child.

Current preventive strategies have mainly focussed on increasing physical activity and improving healthy eating [12]. Despite a trend towards a reduced prevalence of GDM in overweight or obese women [13, 14], there is an urgent need for more well-designed effective lifestyle interventions for the prevention of GDM.

Adopting a healthy lifestyle may be particularly demanding for overweight or obese pregnant women as they are more likely to be less physically fit and have poorer quality diets [15, 16]. Still pregnancy seems a perfect time to intervene and discuss weight management, since women accept their weight and weight gain more than when they are not pregnant [17]. Understanding the beliefs, barriers, and preferences of overweight pregnant women is key for developing effective lifestyle modification programs, but research in Europe is scarce.

Weir and colleagues [18] have conducted an interview study in the United Kingdom (UK), in which they found that healthy eating was often viewed as being of greater importance for the health of mother and baby than participation in physical activity. Also, participants often described how they would wait until the postnatal period to try and lose weight. A wide range of barriers to physical activity during pregnancy were highlighted including both internal (physical and psychological) and external barriers (work, family, time, and environmental). The study participants also lacked access to consistent information, advice, and support on the benefits of physical activity during pregnancy.

As part of a larger European project, we set out to enhance our understanding of beliefs, barriers, and preferences of European overweight and obese pregnant women regarding lifestyle modification in view of prevention of GDM.

## 2. Methods

### 2.1. Study Design

The study was designed as an exploratory two-staged project, applying mixed methods to inform directly the development of a European lifestyle program (Vitamin D and Lifestyle Intervention: DALI project [19]), which will target prevention of GDM in an overweight and obese population. The DALI study is conducted in nine European countries: Austria, Belgium, Denmark, Ireland, Italy, Netherlands, Poland, Spain, and the UK. This study set out to develop and test a suitable lifestyle program across all these countries, since except the UK [18] there are no data regarding preferences, beliefs, and barriers of lifestyle modification. Language difficulties required a pragmatic approach. Therefore the choice was made to start with qualitative interviews in Netherlands and Belgium, conducted by a Dutch speaking person (KvL) educated in health science. Secondly, and based on the interview results, a cross-national questionnaire was performed with overweight and obese pregnant women from six European countries (Austria, Denmark, Ireland, Italy, Poland, and Spain). In the UK, instead of this questionnaire, five more interviews were held, which were based on the questionnaire and the topic guide previously used in Netherlands and Belgium. These interviews were conducted by an English-speaking person (CB).

The study was guided by the Health Action Process Approach (HAPA) model of behaviour change, which builds on social-cognitive theory to help predict (preventive) health behaviour change of individuals at risk [20], and the Motivational Interviewing (MI) framework, which is a collaborative, person-centered form of guiding to elicit and strengthen motivation for change [21]. Special focus is given to (i) risk perception and perceived importance, (ii) barriers and perceived self-efficacy, and (iii) preferences with regard to support in lifestyle modification.

The Institutional Review Board of the VU Medical Center and local ethical committees from the respective centers in the nine countries approved the study (NRES Committee East of England-Norfolk: 11/EE/0221; Medical University of Poznan: 1165/12; UZ KU Leuven: ML7625; VUmc Amsterdam: 2012/400; Hospital de la Santa Creu i Sant Pau, Barcelona, 13/006 (OBS); Medical University of Vienna: 2022/2012 – 1369/2013; Region Hovedstaden Copenhagen: H-4-2013-005; Province of Padua: 4201 × 11; Galway University Hospitals: 7/12).

### 2.2. Study Participants and Recruitment

In both phases of the study women with a prepregnancy body mass index (BMI) ≥ 25 kg/m^2^, which is a risk factor for the development of GDM [22], were randomly sampled. Women either were pregnant or had given birth within the last 12 months. Those that already had given birth were asked about the time while being pregnant, which provided additional data on received information and care around weight management as part of pregnancy care.

For the interviews, women were identified from those attending obstetric services for pregnancy in Amsterdam, Zwolle, or Enschede, Netherlands; Leuven, Belgium; Cambridge, UK. In Belgium and the UK women were recruited by their healthcare professional. In Netherlands women who previously were approached to participate in a lifestyle intervention program [23] were invited. Of the women included from Netherlands, five had actually experienced this lifestyle intervention program and five had previously declined participation. The women who were unable to come to the research center or hospital were interviewed by telephone. In Netherlands and in Belgium 53 women were approached to take part in the current study, and all those who replied positively have been included. We have no data on reasons for lack of willingness to participate. No data exists either on the total number of women invited in the UK.

For the questionnaire, women were identified in the participating obstetric services for pregnancy in Vienna, Austria; Copenhagen, Denmark; Galway, Ireland; Pisa or Padua, Italy; Poznan, Poland; and Barcelona, Spain. In all these countries women were recruited by their healthcare professional. No information exists on the total number of women invited in the different countries.

### 2.3. Data Collection

#### 2.3.1. Phase 1: Semistructured Interview Procedure

A thematic interview guide with predefined questions was used, while giving the participants the freedom to elaborate on a particular subject. Included questions were based on the particular objective of this study and findings from previous studies [16, 18, 24–27]. The interview started with introductory questions concerning the women's experiences with pregnancy and the importance of a healthy lifestyle. Next their beliefs, experiences, perceived barriers, and facilitators regarding a healthy diet and physical activity were investigated, followed by questions about preferred types of support, activities, and mode of delivery of an intervention. A pilot-test of the interview guide was carried out with one pregnant woman (not included in the study), which resulted in minor changes in the wording. The interviews comprised 15 face-to-face interviews and six telephone interviews and lasted between 15 minutes and 120 minutes and were voice-recorded and transcribed verbatim. The interviews were performed by KvL (Netherlands and Belgium) in the period of January 2010–April 2010 and CB (UK) in the period of November 2011–April 2012. After 16 interviews theme saturation was achieved and confirmed with the five final interviews conducted in the UK.

#### 2.3.2. Phase 2: Questionnaire Procedure

In phase two, the topics from the interviews were rewritten as statements in a questionnaire, with response categories on a 5-point Likert scale (strongly agree, agree, neutral, disagree, and strongly disagree) and with space for open ended comments. The questionnaire contained closed and open ended questions in English and was sent to participating obstetricians, midwives, and physicians in Austria, Denmark, Ireland, Italy, Poland, and Spain to gather information from those other European countries. Pregnant women completed the questionnaire in their native language together with their midwife/obstetrician/physician during a consultation, except for Spain where the questions were answered by telephone. Both face-to-face and telephone conversations were audio recorded and the comment responses and open questions were back-translated by the midwife/obstetrician/physician into the English language and sent to Netherlands for analyses. In total 71 questionnaires were completed in the period of July–December 2010.

### 2.4. Data Analysis

The transcripts of the interviews were coded by KvL and analysed according to the framework method of qualitative data analysis [28] using software package AtlasTi 5. The coding for three interviews was independently reviewed by a second researcher (NO), showing high agreement. A few disagreements were resolved by discussion.

All questionnaire data were entered in SPSS (v15.0) (SPSS Inc., Illinois, USA). The answer categories for “strongly agree” and “agree” were combined (referred to as agreed), as well as “strongly disagree” and “disagree” (referred to as disagreed). Descriptive statistics (frequencies and percentages) were used to summarize quantitative data. Chi square statistics were used to explore associations between answers and respondent characteristics. The level of statistical significance was set at *p* < 0.05.

## 3. Results

The majority of the women involved in the interview part of the study had a high level of education and had a European background (Table 1). Fifteen women were interviewed throughout pregnancy (between 16 and 39 weeks of gestational age) and six women were interviewed between 0 and 12 months after delivery. In the questionnaire part of the study women tended to be more equally divided across age and educational level. Sixty-six women were between 6 and 40 weeks of gestational age and five women were between 3 and 4 months postpartum. In both groups about 60% of all the women had another child at home.

The learning from this study, combining interview and questionnaire data, is grouped in three categories, with reference to the HAPA model [20] and MI framework [21]: (i) risk perception and perceived importance, (ii) barriers and self-efficacy, and (iii) preferences with regard to a lifestyle program aimed at assisting in improving physical activity and eating habits in order to manage gestational weight gain. The main results are presented in Table 2.

### 3.1. Risk Perception and Perceived Importance

The most important motivator for a healthy lifestyle was the health of their babies (100%; see Table 2).

*The health of your child is most important, that's my top concern. Of course, my own health as well, but I have my child more often in my mind than myself. (#8, 19 weeks pregnant woman with 2nd child, Netherlands)*

In this study 62% of the women indicated that nobody had ever talked to them about the risks and consequences of GDM (Table 2). Although all of the women were overweight or even obese, only 57% thought they had a high risk of actually developing GDM (Table 2). Some women acknowledged that they would be more proactive in maintaining a healthy lifestyle if caregivers would emphasize the importance of doing so, for instance, by paying attention to their higher risk for diseases and complications caused by their weight.

*There is little time to talk about these things. I was told ‘there is a higher risk for you to develop diabetes' […], but you have to search for information yourself. Nobody ever mentioned to me what it means to have diabetes or gestational diabetes. I think that would help, so that you really grasp the consequences. (#4, delivered 3rd child, Netherlands)*

*I noticed that care providers hardly ever bring up weight issues. I think it is important that general practitioners and midwives give us more guidance in controlling our weight. (#3, delivered 1st child, 4 months postpartum, Netherlands)*

### 3.2. Barriers and Perceived Self-Efficacy

Barriers for women to be physically active during pregnancy may be internal and/or external. Two internal barriers brought forward by our interviewed women and quantitatively scored by those who filled out the questionnaire were experiencing physical complaints (80%) and tiredness (46%) (Table 2).

*In the beginning you are tired, a lot. Just not enough energy …, you come back from work, but are too tired to go out again. (#13, delivered 1st child, 2 months postpartum, Belgium)*

Not having sufficient time (34%) was reported as external barrier (Table 2). Those women with children were more likely to agree that they had too little time to be physically active (47%) compared to nulliparous women (14%) (*p* = 0.002).

* Before my first pregnancy I used to exercise a lot. Then I got pregnant again for a second time real soon and had no time anymore. ‘…' My physical activity is walking at home, the stairs and running after the children. (#15, 16 weeks pregnant woman with 3rd child, Belgium)*

Almost all women (92%) were motivated because they felt better after completing any physical activity (Table 2). In addition, the interviewed women indicated that regular engaging in physical activity with friends or others during their pregnancy supported them in being active.

The interviewees tended not to differentiate between a healthy diet in general and during pregnancy, but they did mention the importance of meeting their unborn child's nutritional needs and the foods that should be avoided during pregnancy, such as soft cheeses and raw meat.

*Ever since I have been pregnant I have made a very very conscious effort to make sure I was doing the right things and eating the right things, because obviously, I'm trying to grow someone (laughs). (#19, 35 weeks pregnant woman with 2nd child, UK)*

Women with children found it less difficult to maintain a healthy diet throughout pregnancy (30%), compared to women without children (64%) (*p* = 0.01). Other frequently experienced barriers to eating healthily mentioned by the interviewed women were having cravings, social gatherings, and being busy.

* Being an example is motivating. It is impossible to take candy and tell your children they can't have it. […] If you do not want your child to drink cola, then you should not be doing it yourself. (#11, 35 weeks pregnant woman with 2nd child, Belgium)*

### 3.3. Preferences for a Lifestyle Program

A program addressing both healthy eating and physical activity was preferred by the women, in which personal risks, consequences, and emotional issues relating to weight and GDM should be addressed.

*Forcing things down people's throats I believe is not the way to go, so actually having someone say do you think this might be the best option, to try to encourage you to choose for yourself, but not forcing it down your neck is a good way of people trying to communicate with you to eat healthily. (#17, pregnant with 6th child, UK) *

Women would like to talk to a health provider, coach, or dietician (86%) and were motivated if another person checked their diet and weight regularly (76%; see Table 2).

All women were generally in favour of being offered multiple choices in terms of time, location (at home or in a hospital), communication channels (face-to-face, telephone, and/or Internet (see Table 2)), and activities (swimming, walking, group fitness/exercises, and cycling).

*I don't know because some people work better in groups some people do not, I am more on my own kind of thing, because sometimes it might get a bit too much, so it depends on the person. I would probably be like one on one. (#21, pregnant with 2nd child, UK)*

The support of the partner is seen as extremely important (91%; see Table 2), underscoring the need to include the partner in the process of behaviour change in pregnant women.

## 4. Discussion

We explored beliefs, experiences, and preferences regarding lifestyle modification during pregnancy among a diverse sample of overweight and obese European women who are at increased risk of developing GDM. The interviews in Belgium, Netherlands, and the UK provided an in-depth analysis and richness on risk perception of GDM, barriers, and facilitators regarding the topics physical activity, healthy eating, and weight control. The questionnaire results across other European countries corroborated our findings from the interviews, providing valuable information on the best European approach to intervene in the lives of overweight and obese pregnant women.

To make changes to ones' behaviour a person should first perceive a necessity to change [20]. Perceived importance of behaviour change is one of the determinants of motivation and strongly driven by risk perception [20]. This may pertain to the woman's own health as well as the health of the unborn baby. All women in our study valued the importance of the health of their unborn child, which is not a unique finding but definitely underscoring the observation that pregnancy is a key time in which women are motivated to live healthily [29].

Consistent with earlier research, in our study pregnant women appear highly receptive to health information and advice during pregnancy [30]. However, professionals do not use this opportunity to discuss the accompanying risk of obesity in pregnancy as they experience it to be a “conversation stopper” [31]. Furthermore, professionals report they do not want to offend their clients by addressing their obesity and the risks involved [32, 33], which could potentially impact negatively on the midwife-woman relationship [33]. Based on an in-depth interview study in obese pregnant women, a clear need exists for training of professionals in nonjudgemental weight counselling and motivational techniques [34]. The need for this motivational training is supported by the observational study of Brown et al. collecting all the verbal and written information provided to first-time pregnant women regarding physical activity, diet, and weight management, which lacked purpose goals within verbal instructions, performance feedback, and specificity and relevance of target goals [35].

The barriers related to physical activity like physical complaints, tiredness, and time mentioned by the women in this study were mentioned by normal weight European pregnant women in previous conducted studies as well [18, 36–39], suggesting that these barriers may apply to the whole pregnant population. So far, studies on the relationship between BMI and exercise during pregnancy are inconclusive [40], although it would be of interest to see if physical complaints differ in magnitude and severity between a normal weight and overweight population.

In our study women who had at least one other child indicated that time constraints made it harder for them to be physically active, which is consistent with other studies in which first-time pregnant women were 1.6–1.9 times more likely to be physically active compared to multipara women [40]. Providing childcare or home-based interventions may prove helpful in this context.

Pregnancy often results in a decline in physical activity levels [41], as reported by the participants in our study. American guidelines suggest that pregnant women who are sedentary prior to pregnancy should build up their activity level to at least 30 minutes of at least moderate activity a day, while already active women should maintain or increase their level up to 30–60 minutes a day [42]. However, prescribing physical activity will only translate into behavioural change if the person is motivated and confident of her own ability to actually make that behavioural change. Self-efficacy is highly related to intention formation [20], weighing the pros and cons of a specific behavioural change. It is found that higher levels of self-efficacy to exercise and to overcome exercise barriers are associated with more leisure time physical activity during pregnancy [43]. Promoting pregnant women's self-efficacy by health counselling is therefore key.

There are many benefits from being more physically active for both maternal and foetal health [44]. Yet, these benefits have not been reported by the women in this study, which suggests a need for an improvement in the quantity and quality of information related to physical activity presented by healthcare professionals [41].

This research indicates on the one hand difficulties for women with children to become physically active, yet on the other hand they experience fewer problems to eating a healthy diet. Eating healthier may be due to their wish to set an example for their children or they may be more knowledgeable regarding healthy food from a previous pregnancy. In intervention development it may be especially valuable to distinguish between first-time mothers and those women who already have children.

Based on the results from this study an intervention program across Europe should primarily be flexible to attend and individually tailored to a woman's personal lifestyle, addressing topics related to weight management, physical activity, and healthy eating. The partner is seen as highly important by almost all women, which is concurrent with earlier research [18, 26, 36]. In addition to partner support, this research suggests that it may be important to extend interventions to family and friends since they might discourage these women from being more active especially in third trimester of pregnancy [41].

## 5. Strengths and Limitations

This mixed method study was conducted in a heterogeneous group of overweight and obese women, making it possible to investigate the views of women living in nine different countries across Europe. This favours the external validity. However, selection bias cannot be excluded, as we have no information available on the nonresponders. There were clear trends and answers were rather comparable across sites, with no obvious differences observed between countries. This would justify the implementation of a similar intervention strategy across these countries. However, we should interpret our findings with caution given the small sample size of this study, which made it impossible to conduct separate analysis by “country.” We further recognize that women's responses might have been different for those “being overweight or obese” or those “currently pregnant or postpartum” women. Additionally the type of experienced barriers across pregnancy may have been dynamic as was found in earlier research [43], although it was not our intention to investigate this, since we wanted to develop an intervention to intervene across the whole pregnancy. Also the data gathering either by telephone call or by face-to-face interview might have had an impact on the results, although we believe that this pragmatic approach led to the inclusion of women who would otherwise not have participated due to time constraints and therefore we see this as a valuable addition to the data collection process. Translation of questions into different European languages and back might have influenced our results, although we found striking consistency in responses across countries. Furthermore, it is a limitation that detailed information regarding the response rate is lacking in most of the countries.

## 6. Conclusion

From our results it would appear that overweight and obese pregnant women overall are motivated to adopt a healthy lifestyle, but we cannot assume all overweight and obese pregnant women have that “readiness to change” [45]. Raising and reinforcing risk awareness combined with promoting self-efficacy warranted special attention, with a prominent role for health professionals [46]. A tailored counselling intervention attuned to the stage of behaviour change [20] and taking into account barriers related to parity might be beneficial. In the end, this study led to the development of the DALI intervention program, in which lifestyle coaches in addition to the usual care women receive will provide risk communication and individual lifestyle counselling on lifestyle behaviours such as weight management, physical activity, and healthy eating. A detailed description of the developed intervention is written elsewhere [19]. Further research will demonstrate if addressing the issues that emerged from this study can help European overweight and obese women successfully prevent GDM.

## Figures and Tables

**Table 1:** Characteristics of participating overweight and obese pregnant women (*N* = 92).

Characteristic	Number of participants, interview (total *N* = 21)	Number of participants, questionnaire (total *N* = 71)
Age		
Younger than 30 years	8 (38%)	23 (32%)
Between 30 and 35 years	10 (48%)	23 (32%)
Older than 35 years	3 (14%)	25 (35%)
Educational level		
Academic graduate	8 (38%)	21 (30%)
Higher education graduate	8 (38%)	3 (4%)
High-school graduate	2 (10%)	26 (37%)
Vocational training	2 (10%)	11 (16%)
Primary school	1 (5%)	9 (13%)
Unknown	—	1 (1%)
Country of birth		
Netherlands	10 (48%)	—
Belgium	6 (29%)	—
United Kingdom	5 (24%)	—
Italy	—	20 (28%)
Spain	—	10 (14%)
Ireland	—	10 (14%)
Poland	—	10 (14%)
Austria	—	11 (16%)
Denmark	—	10 (14%)
		
Both parents born in Europe	19 (90%)	59 (83%)
One of the parents born elsewhere	2 (10%)	12 (17%)
Parity		
Nulliparous	8 (38%)	28 (39%)
Parous	13 (62%)	43 (61%)
		
Pregnant	15 (71%)	66 (93%)
Already given birth	6 (29%)	5 (7%)
Experience with GDM prevention program		
None	16 (76%)	71 (100%)
Yes	5 (24%)	—

**Table 2:** Interview questions and corresponding questionnaire statements and answers.

Interview questions	Interview answers (*N* = 21)	Statements questionnaire	% agreement (*N* = 71)	Remarks given by participants (*N*)
Risk perception and perceived importance				
Are you familiar with GDM, the consequences and risks associated with it?	*Most are informed but not extensively*	Nobody ever talked about the consequences of gestational diabetes with me	62	*Would you have appreciated if someone had done so?* Yes (46); No (4); Only if I had developed GDM (3)
How high do you estimate your own risk for developing GDM?	*Different risk perceptions were reported*	I think I have a high risk for developing GDM	57	*Why do you think so?* (over) weight (26); (family) history (G)DM (20); Unhealthy lifestyle (6); My doctor said so (4)
				*Why don't you think so?* Healthy diet and enough exercise (8); Normal test results (7); No (family) history DM/problems (7); Don't know anything about GDM (3)
How important is GDM prevention to you?	*Almost all women think this is important*	I would go to great length to prevent GDM	92	*How could you prevent GDM?* Healthy nutrition (59); More physical activity (32); Lose weight (7)
How important is your health for you? How did this change during your pregnancy?	*All indicate health of baby most important*	A healthy lifestyle is very important at this moment	94	*Is this different from before you were pregnant?* No (31) Yes (32) Yes, but I can do less (back pain) and I am eating more (5)
	*Important to eat the correct food*	Already during pregnancy I feel responsible for the health of my baby	100	No remarks
How did your weight change over the past years and how does this affect you? Do you mind people telling you that you are overweight?	*Most were struggling with their weight for years, but prefer a more advocate approach from health provider*	I would not mind health providers telling me I am overweight	79	No remarks

Barriers and perceived self-efficacy				
What do you think is a healthy diet during pregnancy? Is this difficult for you to follow? What makes this difficult?	*A healthy diet in pregnancy does not differ from a diet in general.*	It is not difficult to maintain a healthy diet during pregnancy	49	*In which situations is this difficult?* Cravings/hungry (22); Social occasions (10)
	*Being busy, social gatherings, cravings and the notion to eat for two made it harder than usual*	In my surroundings it is common for pregnant women to eat and snack more than usual	62	*Is this extra difficult?* no (24); yes (17); sometimes (3)
How physically active were you before you became pregnant, how did this change during your pregnancy?	*Most participants stopped dangerous sports and participated in less intensive activities*	I have not changed my exercise/physical activity habits during pregnancy	38	*What has changed?* Less physical activity (walking, running, cycling, swimming) (25); More physical activity (walking, swimming) (12) Changed the type of activity (5)
What makes it hard for you to become or stay physically active during your pregnancy? And what will make you stop?	*Tiredness, being to busy and physical problems are the most frequently mentioned barriers which make it harder than usual to stay physically active*	I will stop with physical activity when I develop physical complaints	80	*What will make you stop?* Complaints of pain (hard belly, back-, pelvic-, abdomen-, muscle-, leg pain) (30); Tiredness/exhausted (11); Bad for baby (9); Doctors advice (6); Heart problems/headache/migraine (5); Blood loss (2)
		I am too tired to be physically active	46	No comments
		I have too little time to be physically active	34	*What other practical barriers keep you from physical activity?* Other children/childcare (19); Motivation/Dislike of physical activity/Laziness (9); Work (6); Costs (6)

Preferences				
How should we encourage pregnant women to take part in a prevention program?	*Most women thought guidance, weight control and support research were reasons to participate in a prevention program*	I find aiming for weight control in pregnancy more appealing than the prevention of gestational diabetes	37	*In favour of weight control:* Both equally important (1); Weight control prevents GDM (4) Weight is a bigger problem (4); Weight control is simpler (2)
				*In favour of GDM:* Both equally important (15); GDM more important (9); GDM more dangerous for baby (3); More afraid of developing GDM (2); Focusing on GDM affects weight (2)
How should an intervention program look like? Do you have preference regarding guidance? Communication channels? What should definitely be included?	*Talking about weight problems and addressing physical activity and healthy eating should be included in a prevention program*	It motivates me if another person checks my diet and my weight regularly	76	No remarks
		I would like to talk to a health provider, coach or dietician about my weight problems	86	No remarks
What motivates you to become physically active and eat more healthily?	*Support from partner and regular appointments with friends/others*	I feel better if I am physically active	92	No remarks
	*Thinking of the consequences and feeling good afterwards*	It is important for me that my partner supports me	91	No remarks
Would you need help with healthy eating and physical activity in pregnancy and what kind of help would you appreciate?	*Dietary advice from a dietician would be appreciated, regarding what (not) to eat and weight management*	During pregnancy I would appreciate having personal support for having a healthy diet	71	*How?* Face-to-face (dietician, physician, obstetrician) (39); Internet (12); Telephone (11)
What were reasons for you to seek help regarding weight related issues?	*They knew what to do regarding weight control in general but not specifically during pregnancy*	I know what I have to do to lose weight in general, but I don't know how to control my weight during pregnancy	44	*Why is it different for you in pregnancy?* Responsible for health of baby (8); More hunger (7); Difficult to do physical activity (6); Difficult to follow a diet (4); You don't notice, because of growth of baby (3); I don't know (3)
		I know where pregnant women can get help with weight control in my surrounding/neighbourhood	30	No remarks

DM refers to diabetes mellitus; GDM refers to gestational diabetes mellitus.

DM/problems refers to no family history of DM or problems with DM.

## Abbreviations

BMI: Body mass index
GDM: Gestational diabetes mellitus
HAPA: Health Action Process Approach
MI: Motivational Interviewing
UK: United Kingdom.
